# Lipid metabolism in tumor immunology and immunotherapy

**DOI:** 10.3389/fonc.2023.1187279

**Published:** 2023-05-02

**Authors:** Lisa K. Duong, Halil Ibrahim Corbali, Thomas S. Riad, Shonik Ganjoo, Selene Nanez, Tiffany Voss, Hampartsoum B. Barsoumian, James Welsh, Maria Angelica Cortez

**Affiliations:** ^1^Department of Radiation Oncology, The University of Texas MD Anderson Cancer Center, Houston, TX, United States; ^2^Department of Medical Pharmacology, Cerrahpasa Medical Faculty, Istanbul University-Cerrahpasa, Istanbul, Türkiye

**Keywords:** lipids, lipid oxidation, immunotherapy, cancer, immune cells

## Abstract

Lipids are a diverse class of biomolecules that have been implicated in cancer pathophysiology and in an array of immune responses, making them potential targets for improving immune responsiveness. Lipid and lipid oxidation also can affect tumor progression and response to treatment. Although their importance in cellular functions and their potential as cancer biomarkers have been explored, lipids have yet to be extensively investigated as a possible form of cancer therapy. This review explores the role of lipids in cancer pathophysiology and describes how further understanding of these macromolecules could prompt novel treatments for cancer.

## Introduction

Lipids, one of four broad classes of macromolecules in living organisms, are hydrophobic organic molecules with a diverse variety of important functions in many cellular processes. Some notable functions related to cancer progression include the involvement of lipids in cellular signaling, energy storage, and inflammatory and immune responses (1) . Accumulation of lipids in the tumor microenvironment (TME) has been shown to promote immune evasion and inflammation (2, 3), and lipids are an important source of energy for rapidly proliferating cells (3).

**Figure 1:**
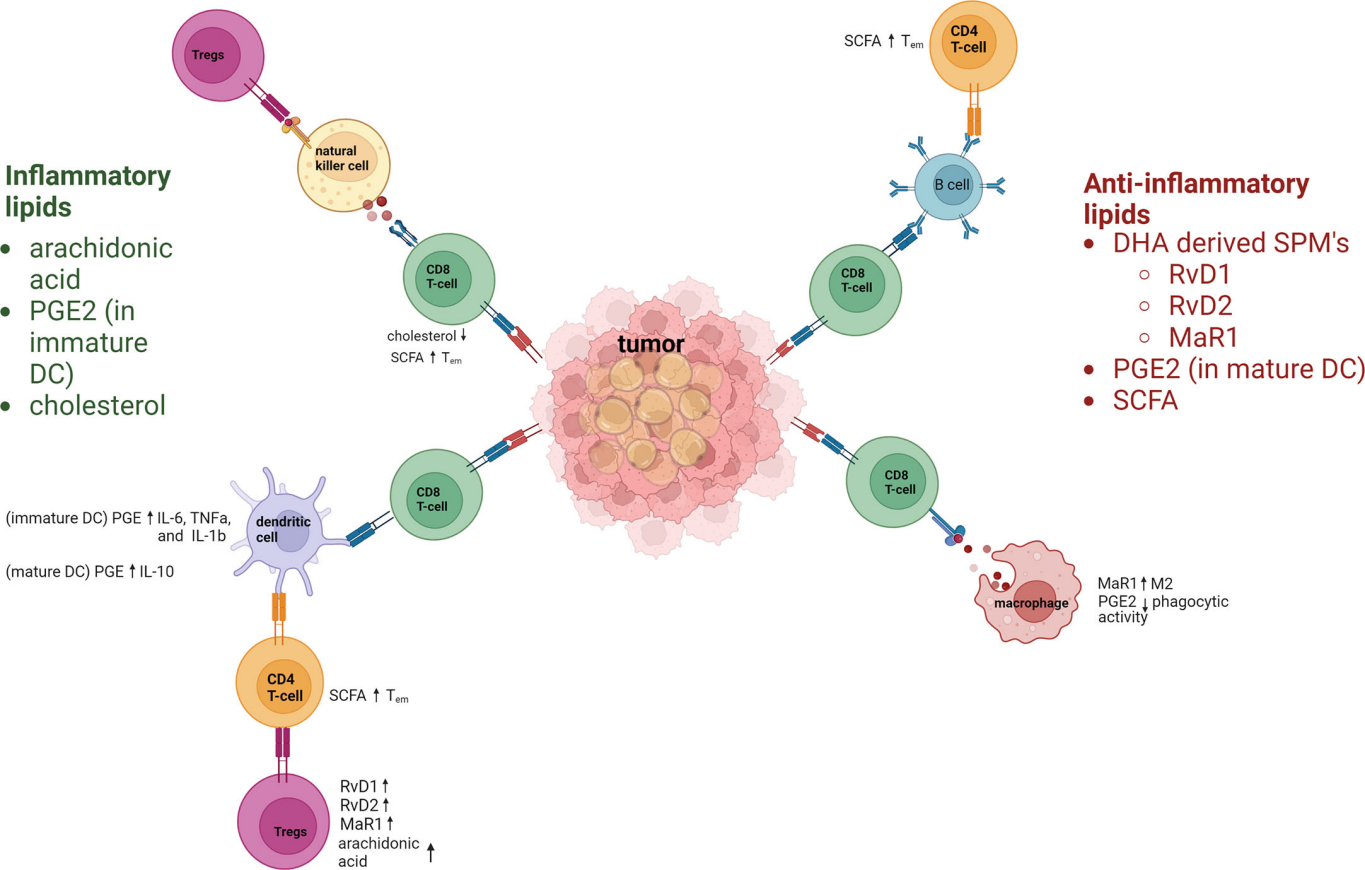
Inflammatory and anti-inflammatory lipids regulation of the immune response. Lipids have key roles in regulating immune–inflammatory responses. Inflammatory lipids include the polyunsaturated fatty acid arachidonic acid, PGE2, and cholesterol. Arachidonic acid hampers immune responses by increasing Tregs. PGE 2 increases inflammatory cytokines in immature DC. Cholesterol contributes to CD8^+^ T-cell dysfunction due to increases endoplasmic reticulum stress. Anti-inflammatory lipids include the specialized proresolving lipid mediators (derived from docosahexaenoic acid [DHA]) resolvins D1 and D2 (RvD1, RvD2), and maresin-1 (MaR1), all of which act *via* dendritic cells (DCs) to upregulate T regulatory cells (Tregs). PGE2 and short-chain fatty acids (SCFA) are also anti-inflammatory lipids that regulate immune cells. SCFAs increase the effector memory phenotype of T cells. Macrophages are polarized towards M2 by MaR1, and macrophage phagocytic activity is downregulated by PGE2. PGE2 also upregulates IL-10 in mature DC.

Lipids also affect the immune system and its components in a variety of ways (**Figure 1**). Abnormal lipid accumulation in tumors correlates with T-cell dysfunction, T-cell exhaustion, increased proportions of regulatory T cells (Tregs) (4, 5) and memory T cells, and increased T-cell recall responses (6). Lipids also influence macrophage functions, in some cases causing increased plasticity (7, 8) and in others leading to decreases in macrophage differentiation that can subsequently enhance tumor growth (8, 9). The presence of lipid droplets in tumors has also been linked with the presence of natural killer (NK) cells and increased metastasis (10). Similarly, triglyceride accumulation in dendritic cells (DCs) has caused downregulation of antigen presentation and increased immune evasion.

This review focuses on the functions of lipids in the immune system and their effects on cancer progression and metastasis. Specifically, this review covers how lipids affect T cells, macrophages, NK cells, and DCs, as well as their roles in immunotherapy, cell cycling, and cancer metastasis, all as a means of prompting interest in novel treatment strategies based on this macromolecule. Also reviewed is the controversial evidence supporting the potential of lipids to serve as biomarkers in cancer treatment and early evidence of their activity in cancer progression.

## Lipids and lipid oxidation in immune cells

### T cells

T lymphocytes are an indispensable component of the adaptive immune system, as they mount cell-mediated responses to fight pathogens as well as tumor cells. T cells are of three broad types: helper T cells (CD4^+^), cytotoxic T cells (CD8^+^), and suppressor T cells (e.g., CD4^+^ Tregs). Like all cells of the immune system, T cells are created in the bone marrow; uniquely, however, they mature in the thymus. The maturation process involves negative selection, in which T cells that are activated by “self” proteins undergo apoptosis. Once the T cells leave the thymus, they are considered to be mature but naïve, meaning they have not encountered corresponding antigens and will remain in the G0 phase of the cell cycle until they do so. The differentiation processes and the effector functions of T cells are fundamentally tied to cellular metabolism. During differentiation, T cells undergo metabolic reprogramming to meet the divergent energy needs of each cell type. This means that understanding the functions of cells at each stage of differentiation will help to reveal the metabolic pathways that are upregulated (11).

For example, naïve T cells require energy to migrate throughout lymphoid organs with little need for biosynthesis (as they are not proliferating), and resting T cells generate adenosine triphosphate (ATP) as an energy source by fatty acid oxidation (FAO). Activation of T cells leads to their becoming metabolically reprogrammed to a state of anabolic metabolism in which lipid biosynthesis is upregulated (as opposed to lipid oxidation) to allow the cells to divide and proliferate (12). Activated T cells become proliferative upon encountering infectious agents, and some proportion of those proliferating antigen-specific T cells develop into memory T cells, which persist even after contraction of the antigen-specific effector cells. Like naïve T cells, memory T cells use catabolic metabolism in the form of FAO in addition to oxidative phosphorylation to meet their metabolic demands and fulfill their functions. FAO is crucial for the transition of activated CD8^+^ T cells into memory T cells; indeed, one study showed that altered expression of genes that regulate FAO was found to correlate with a defect in memory T cell generation (13). The same study also showed that pharmacologic modulation of FAO could enhance CD8^+^ T-cell development after vaccination (13). These and other studies have established a link between lipid metabolism and cellular longevity. CD4^+^ Tregs, which express the protein Foxp3, are a subset of T cells that are crucial for self-tolerance. Previous studies have linked the pool of colonic Tregs in the gut with the concentration of short-chain fatty acids (FAs) produced by gut bacterial fermentation (14). Follow-up studies showed that these short-chain FAs expanded Tregs by suppressing JNK1 and p38 pathway signaling (15), which is crucial for intestinal homeostasis. Short-chain FAs derived from the microbiota also affect CD8^+^ memory T cells. As noted earlier, FAO is important for the formation of memory T cells but microbiota-derived short-chain FAs in particular were required for optimal recall responses upon antigen re-encounter, as was observed in one study by memory-cell defects in germ-free mice (6). That same study showed that the memory T cells in mice consuming a high-fiber diet, which increases circulating levels of the short-chain FA butyrate, had significantly higher numbers of effector cells than did control mice (6). These findings confirm that metabolic substrate availability in the environment has a profound influence on the differentiation and function (or dysfunction) of T cells. Another example of this can be seen in the TME, which is often rich in cholesterol and FAs (4). Lipid uptake by Tregs in the TME depends largely on FA translocase (CD36), and TregCD36^–/–^ mice showed a profound loss of intratumoral Tregs (16). On the other hand, increased CD36 expression on CD8^+^ tumor-infiltrating lymphocytes (TILs) caused by lipid accumulation in the TME was found to correlate with progressive T-cell dysfunction, thought to be caused by uptake of oxidized low-density lipoprotein by CD36, which induced lipid peroxidation and downstream activation of P38 (4). Moreover, CD36-mediated uptake of FAs by CD8^+^ TILs in the TME led to ferroptosis and reduced the production of cytotoxic cytokines (17).

T-cell proliferation is also affected by long-chain polyunsaturated fatty acids (PUFAs) (18). The immune response is downregulated as n-3 PUFAs become incorporated within the lipid rafts of the cellular membrane (19), causing a decrease in pro-inflammatory molecules such as PGE2, TXB2, and LTB4 and a decrease in inflammatory cytokines (20). Arachidonic acid, an n-6 PUFA, has been linked with decreased production of interleukin (IL)-10 and interferon-gamma (IFNy) and upregulated Treg production, which ultimately dampens the T-cell immune response (18).

Other compounds that affect T-cell regulation are the so-called specialized proresolving lipid mediators (SPMs) derived from the FA docosahexaenoic acid (21). In one study, treating human T cells with the SPMs resolvin D1 (RvD1), RvD2, and maresin 1 (Mar1) led to downregulated production of the inflammatory cytokines tumor necrosis factor-alpha (TNFα) and IFNy by CD8^+^ T cells, as well as downregulated IL-17 in CD4^+^ cells. Treatment of CD4^+^ naïve T cells with SPMs also prevented those cells from differentiating into T helper 1 (TH1) and T helper 17 (TH17) cells (21). These same three SPMs also enhanced immunosuppression by increasing the number of Foxp3^+^ Tregs relative to control conditions (21).

Cholesterol and cholesterol derivatives also affect T-cell functions in the TME. In one study, the presence of cholesterol in the TME and in TILs was positively associated with upregulated expression of the immune checkpoint molecules PD-1, 2B4, TIM3, and LAG3 by T cells and T-cell exhaustion (5).

### Macrophages

Macrophages are another important component of the innate immune system with roles in antigen presentation, microbial killing, and regulation of the inflammatory response (22). One of the most salient features of macrophages is their plasticity; they have been shown to activate into different polarized states based on specific microenvironmental conditions and signals (23). Broadly, M1 (classically activated) macrophages mediate pro-inflammatory and antitumor immune responses, whereas M2 (alternatively activated) macrophages are generally understood to mediate anti-inflammatory and pro-tumor immune responses (24). Macrophages that infiltrate tumors, i.e., tumor-associated macrophages (TAMs), can differentiate into either of these subtypes and interact with tumor cells through a variety of signaling molecules (25). Notably, TAMs are the most abundant immune cell in the TME, and they regulate a multitude of pro-tumor effects including angiogenesis, metastasis, and immune evasion (8, 26, 27). Although TAMs also exhibit a variety of metabolic alterations, their reprogrammed lipid metabolism has particularly important effects on their activity.

Generally, increased lipid accumulation in TAMs is positively associated with their differentiation and function, especially because FAO is responsible for the downstream transcriptional regulation of several genes pertinent to TAM activity (7, 8). By extension, several studies have shown that lipid accumulation in TAMs corresponds with tumor progression in a variety of cancer types (28). In gastric cancer, lipid accumulation in TAMs resulted in the upregulated expression of phosphoinositide 3-kinase (PI3K) -γ, which induced M2-like polarization (29). Single-cell RNA sequencing of a subpopulation of TAMs in a mouse model of lung metastases from mammary tumors identified several clusters of macrophages, among them lipid-associated macrophages; these lipid-associated cells were present in greater numbers than in nontumor-bearing controls (30). In human hepatocellular carcinoma, lipid-associated macrophages were found to express TREM2 (a protein with immunosuppressive effects), which correlated with Treg recruitment and poor prognosis (31). Evidently, lipid-loaded TAMs have been broadly investigated for their pro-tumor effects, but their metabolic profiles may offer deeper insights into how they can specifically remodel the tumor immune microenvironment.

Notably, M1 macrophages primarily use glycolysis to produce energy, whereas M2 macrophages predominantly rely on FAO (32). Several studies have been conducted to clarify how FAO specifically regulates TAMs, but they have produced conflicting findings. For example, in one study, inhibiting FAO in TAMs was found to block the pro-tumor effects of M2-like TAMs in a hepatocellular carcinoma model (33), and in another, the chemical inhibitor of FAO etomoxir was found to prevent colon cancer–associated macrophages from polarizing into the M2 subtype (34). In contrast, another study used genetic ablation of carnitine palmitoyltransferase-2 (CP2) to inhibit FAO in macrophages, but those macrophages retained their M2-like markers (35). Some studies have investigated specific pathways or intermediates in lipid metabolism for their relevance to TAM functioning. For example, studies of the role of peroxisome proliferator-activated receptor gamma (PPARγ) in TAM activity have had contradictory results. A deficiency of receptor-interacting protein kinase 3 in hepatocellular carcinoma models inhibited PPAR cleavage, which increased FAO and induced M2 polarization (36). On the other hand, another group found that a binding event between truncated PPARγ and medium-chain acyl-CoA dehydrogenase in mitochondria led to the inhibition of FAO, the accumulation of lipid droplets, and the subsequent differentiation of TAMs. These investigators reasoned that inhibiting the caspase-1-catalyzed cleavage of PPARγ and promoting FAO may actually exhaust lipid droplets, reduce TAM differentiation, and attenuate tumor growth (8, 9). In short, further research is needed to clarify the details of how FAO acts to regulate TAM activity,

Interactions between TAMs and cancer cells are known to be regulated by monoacylglycerol lipase (MGLL), a key enzyme involved in the metabolism of triacylglycerol. One study involving colon cancer models found that a deficiency of MGLL resulted in lipid overload in TAMs and specifically promoted CB2/TLR4-dependent macrophage activation, polarizing TAMs into an M2-like phenotype and suppressing the activity of CD8^+^ T cells in the TME (37). On the other hand, overexpression of MGLL by cancer cells can promote the generation of free FAs, which are an important nutrient source for tumors. Another research group used a nanoplatform to simultaneously block MGLL activity and suppress CB2 expression, which reduced free FA generation and repolarized TAMs into the M1 phenotype (38). More broadly, the metabolism of long-chain FAs, particularly unsaturated FAs, has been shown to promote the immunosuppressive phenotype of TAMs. These findings suggest that enriched lipid droplets may be optimal targets for reversing immunosuppression and enhancing antitumor effects on a metabolic level (34).

TAMs exhibiting altered lipid metabolism can also influence tumor progression through specific molecular factors. One group screened TAM subpopulations among colorectal cancer cells and found that TAMs with lower expression of abhydrolase domain-containing 5 (ABHD5), a coactivator for adipose triglyceride lipase, had higher levels of reactive oxygen species and matrix metalloproteins, which facilitate invasiveness (39). TAMs with enhanced lipid uptake have also been shown to express higher levels of genes for pro-tumor molecules such as *Arg1*, *Vegf*, and *Hif1a* (7, 40). A broad range of lipid metabolites in the TME are also responsible for regulating TAM functioning. As an example, 27-hydroxycholesterol (27HC), a primary metabolite of cholesterol, was found to be highly expressed in TAMs and positively correlated with breast cancer metastasis (41). 27HC was also shown to mediate IL-4-induced M2 macrophage polarization and promoted the recruitment of immunosuppressive monocytes (42). Prostaglandin E2 (PGE2), a mediator of inflammation, has been extensively researched for its multifaceted effects on macrophage activity in cancer. In bladder, nasopharyngeal, and melanoma cancer models, PGE2 has been implicated in promoting the differentiation of myeloid-derived suppressor cells, inhibiting the phagocytic activity of TAMs, and enhancing angiogenesis (40, 43–45). Another metabolite, 5-lipoxygenase, was highly produced by hypoxic ovarian cancer cells and promoted TAM infiltration via upregulation of MMP-7 (46). In murine melanoma models, β-glucosylceramide induced an endoplasmic reticulum stress response, triggering a STAT3-mediated signal cascade that promoted the expression of immunosuppressive genes and supported a pro-tumor phenotype in TAMs (47).

As noted earlier, SPMs can also influence macrophage function. The SPM MaR1 acts as a ligand for retinoic acid–related orphan receptor alpha (RORα), which increases macrophage M2 polarity (48). RORα is a nuclear receptor that regulates inflammatory pathways and lipid metabolism in cells (49). Activation of RORα by MaR1 also decreases M1 polarization and upregulates anti-inflammatory cytokines (48). Corroborating findings with human monocyte lines further support a role of RORα in inflammation; specifically, knocking out RORα in these cell lines led to upregulation of TNF and IL-1B, and RNA sequencing showed that RORα knockout led to activation of cells similar to M1 macrophages (49). SPMs seem to circumscribe or localize inflammation to prevent the development of chronic inflammation (21, 50). Collectively, the various lipid metabolites present in the TME dynamically influence TAM activity and may represent potential therapeutic targets for cancer on an immunological basis.

### Natural killer cells

NK cells are part of the innate immune system that are broadly understood to control tumors and various microbial infections by mitigating the spread of the invading agents and subsequent tissue damage (51). In the context of cancer, NK cells can kill target cells directly through the secretion of perforins and granzymes, which is a hallmark of their cytotoxic activity. They can also produce a host of cytokines and chemokines that facilitate an antitumor immune response (52). However, the phenotype of naive NK cells, including the expression of several activating and inhibiting receptors, can be significantly altered by malignant cells. Tumor-associated natural killer (TANK) cells display these altered phenotypes, resulting either in functional anergy or reduced cytotoxicity (53). For instance, in tumor specimens from patients with non-small cell lung carcinoma, intratumoral NK cells exhibited reduced NK-cell receptor expression, impaired degranulation capacity, and decreased IFN-γ production (54). Similar observations in patients with colorectal cancer further implicate TANK cells in cancer progression (55). TANK cells display a phenotype that mechanistically explains many of their pro-tumor activities; however, the complex interaction between the TME and TANK cells also raises questions about the interplay between TANK cell metabolism—specifically, lipid metabolism—and cancer. Although lipid metabolism in the context of the tumor immune microenvironment has been extensively studied, its specific role in regulating TANK activity remains poorly understood.

One emerging focal point for investigations of lipid metabolism in NK cells is mammalian target of rapamycin (mTOR), a serine/threonine kinase with a central role in signaling lipid metabolism in NK cells, particularly those stimulated by IL-15 (56). One study found that continuous treatment of NK cells with IL-15 exhausted their spare respiratory capacity via FAO reduction and resulted in reduced tumor control; however, treating these NK cells with an mTOR inhibitor rescued their functioning (57). Moreover, obesity was found to result in PPAR-driven lipid accumulation in NK cells, and the administration of FAs along with PPARα/δ agonists (i.e., mimicking obesity) blocked mTOR-regulated glycolysis. Consequently, NK cells trafficked less cytotoxic machinery to the NK cell–tumor synapse and exhibited decreased antitumor activity (58).

Although few studies have directly examined the effects of altered lipid metabolism on TANK activity, one key investigation in the field of perioperative immunology characterized the effect of NK-cell lipid accumulation on postoperative metastasis. That study of both preclinical murine models and human colorectal cancer patient samples revealed that lipid accumulation in NK cells contributed to metastasis compared with controls (10). In the murine models, the scavenger receptors MSR1, CD36, and CD68 (all crucial for intracellular lipid transport and uptake) were all significantly upregulated (10, 56). Also, the lipid-laden TANK cells displayed profoundly reduced tumor-killing ability both *ex vivo* and *in vivo*. The human specimen studies further demonstrated accumulation of FAs in NK cells from 1 to 3 days after surgery; these lipid-laden TANK cells also expressed higher CD36 levels and reduced granzyme B and perforin expression (10). Collectively, the findings from this study suggest that lipid accumulation and dysregulated lipid metabolism in TANK cells participate in facilitating metastasis. Nevertheless, further research into specific lipid metabolic alterations in TANK cells, and research into the interactions between certain lipid metabolites (e.g., PGE2, 27HC) in the TME and TANK cells, is needed to better understand the complex relationship between TANK cells and cancer.

### Dendritic cells

Dendritic cells (DCs) are another type of innate immune cells with key functions in antigen presentation that subsequently connect the innate and adaptive immune systems. The three general types of DCs are plasmacytoid DCs, conventional DCs, or monocyte-derived inflammatory DCs (59). Plasmacytoid DCs specialize in antiviral immunity and create high levels of type I IFNs. Conventional DCs are efficient in antigen presentation and support helper T cells. Monocyte-derived DCs are involved in antigen presentation in cases of infection, inflammation, and cancer and have key roles in cancer immunotherapy; monocyte-derived DCs are also affected by lipid accumulation (59).

Findings from one study of a mouse ovarian cancer model showed that abnormal lipid accumulation led to impairment in antigen presentation by DCs (60). Moreover, this model was also notable for accumulation of lipid peroxidation products, which in turn led to endoplasmic reticulum stress protein folding and subsequent activation of X-box-binding protein 1 (XBP1). Upon isolation of DCs from mice with tumors and healthy mice, cross-presentation of antigens was found to be downregulated in the mice with tumors, and accumulation of lipid droplets led to activated XBP1 (60). Other evidence has implicated PGE2 in both downregulation and upregulation of the immunoregulatory activity of DCs, depending on their stage of maturation (61).. Immature DCs have pro-inflammatory effects in the presence of PGE2, resulting in upregulation of IL-6, TNFα, and IL-1β. In mature DCs, PGE2 leads to IL-10 production, which has anti-inflammatory effects. PGE2 can also inhibit the release of the inflammatory cytokines CCL3 and CCL4 in DCs, which results in downregulation of activated DCs (61).

**Figure 2:**
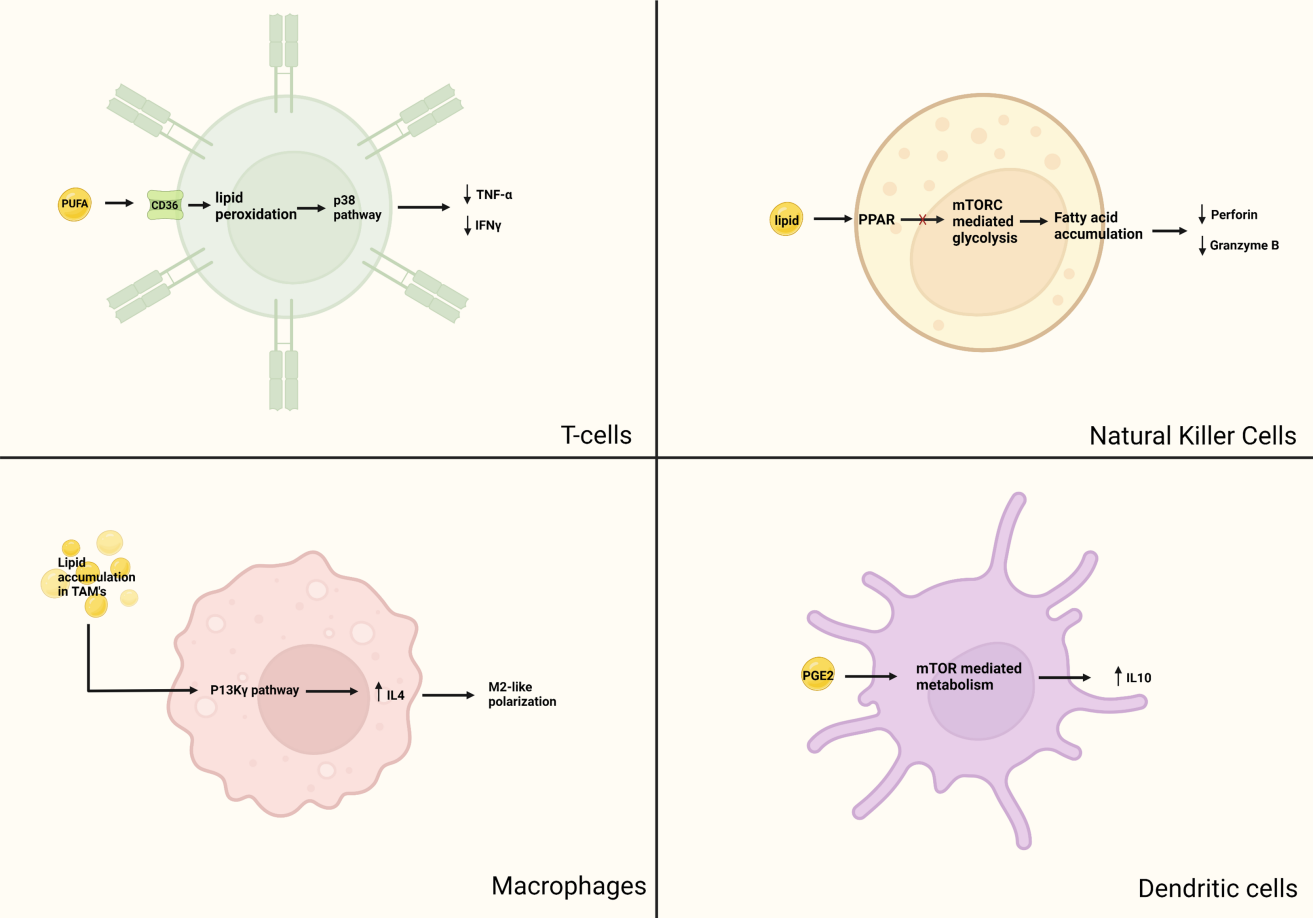
Pro-tumor aspects of lipid metabolism in immune cells. Although lipids are essential for cellular functions, there has been evidence that anormal lipid accumulation can lead to immunosuppression. In T-cells, intake of lipids mediated by CD-36 leads to lipid peroxidation, subsequently causing downstream activation of the p38 pathway. This causes ferroptosis and decreased production of cytotoxic cytokines TNF-α and IFNγ. In macrophages, lipid accumulation in TAM causes upregulation of the P13K-γ pathway, leading to IL-4 induced M2 macrophage polarization and recruitment of immunosuppressive monocyte. In NK cells, obesity has been shown to induce PPAR mediated lipid accumulation and inhibition of mTOR mediated glycolysis. Lipid laden TANK have reduced expression of granzyme B and perforin. Mature DCs have a glycolytic metabolic pathway regulated by mTOR. PGE2 activates mTOR mediated promotion of anti-inflammatory IL-10 production.

Lipid accumulation in DCs has also been noted in mouse models of EL-4 lymphoma stained with the lipid marker BODIPY 493/503 (62). T-cell proliferation in mice with normal-lipid-bearing DCs was compared with that of mice with high-lipid-bearing DCs; the high-lipid DCs showed lower binding affinity with antibody and reduced antigen presentation compared with the normal-lipid DCs. Conversely, manipulation of lipid regulation in tumor cells by using the acetyl-CoA carboxylase inhibitor 5-(tetradecycloxy)-2-furoic acid restored the ability of DCs to stimulate T cells, which led to increased antitumor activity (62). In another model of radiation-induced thymic lymphoma, triacylglycerol serum levels were found to be higher than in control mice (63), which led to decreases in the secretion of IL-12p40, IL-1, and IFN-y by the DCs, thereby downregulating their antigen-presenting function. In another study of specimens from patients with lung cancer, BODIPY 650/665 fluorescence staining revealed elevated triglyceride accumulation, particularly in patients with stage III or IV lung cancer (64). Evaluation of the mixed lymphocyte reaction in these samples (in which T cells are incubated with antigen-presenting cells such as DCs showed that the reaction was weakest in samples from patients with stage IV lung cancer, and that this low level correlated with higher triglyceride levels in the DCs (64). Lipids, therefore, can represent a candidate for immunotherapy targets. The pro-tumor effects of lipids on immune cells are summarized in **Figure 2**.

## Lipids and lipid oxidation in cancer cell proliferation and survival

The uncontrolled proliferation of cancer cells necessitates accumulation of a significant quantity of lipids not only for energy but also to make up the membranes and organelles of these cells. These lipids can be acquired from exogenous sources or synthesized endogenously through lipogenic pathways (65, 66). Understanding how lipids affects tumor cell growth and cell death provides further insight as to how this broad class of biomolecules can be used as a target in cancer treatments.

Cancer that develops in areas of the body with large adipocyte stores tends to have higher amounts of circulating FAs; this higher circulating FAs level plus the nearby adipose tissue influence the metabolism of the cancer (65). As noted previously in this review, depletion of glucose stores during rapid proliferation and growth of tumors leads to areas of nutrient deprivation within those tumors, meaning that TILs rely on oxidative phosphorylation to maintain their energy levels and effector functions (67). When oxygen supplies are limited, the expression of hypoxia-inducible factor 1α enhances glycolysis (65). A lack of both oxygen and glucose may further shift the metabolic profile of TILs to increased FA uptake and catabolism to maintain effector function, with the balance between FAO and ketone body metabolism depending on the extent of oxygen deprivation (67).

As discussed throughout this review, although FA oxidation is a highly efficient form of ATP generation for cancer cells, lipids can also influence proliferation and migration in ways other than providing an energy source (65). As an example, cancer-cell proliferation can also be enhanced by cancer-associated fibroblasts, which transfer lipids to cancer cells through ectosomes. Other examples focus on the interactions between breast tumor cells and lipids, given the large amounts of adipose tissue surrounding breast tumors, with one group studying the “parasitic” relationship of breast cancer cells with adipocytes and lipid stores. That study showed that co-culturing breast cancer cells with adipocytes led to activation of lipolysis within the adipocytes, resulting in the release of FAs into the extracellular space that are then consumed by the cancer cells, fueling both the proliferation and migration of the cancer cells (68). Breast cancer cells have also been shown to respond to the lipolysis of adipose cells by increasing their expression of carnitine palmitoyltransferase 1A, which is the rate-limiting enzyme for long-chain FA transport into the mitochondria for FAO (69, 70). Activation of adipocytes by the nearby cancer cells also leads to the secretion of higher levels of proinflammatory cytokines such as IL-6 (71).

Cancer cells also take up lipids and their building blocks (FAs) to fuel their dissemination and resistance to therapy (72). One way in which this occurs is by the FA scavenger receptor CD36, which can bind and internalize long-chain FAs, lipoproteins, thrombospondin-1, and other pathogen-associated molecules (73).

Lipid metabolism can also induce cell death by changing the permeability of the cell membrane and by activating various enzymes involved in cell death, such as caspases. Changes in membrane permeability also influence ferroptosis (72), a type of cell death that is induced by the iron-dependent peroxidation of polyunsaturated FAs in membranes (72). The process of ferroptosis breaks down membrane integrity, which leads to the death of the cell. Ferroptosis is triggered in iron-rich environments such as blood and in cancer cells, which thus must regulate their membrane lipid composition to survive during dissemination. Although increased membrane lipid saturation can lead to endoplasmic reticulum stress and apoptosis, membrane lipids that contain large amounts of polyunsaturated FAs are more likely to sensitize the cells to lipid peroxidation and ferroptosis (73).

## Lipid metabolism in cancer progression and angiogenesis

### Overview of lipid metabolism

**Figure 3:**
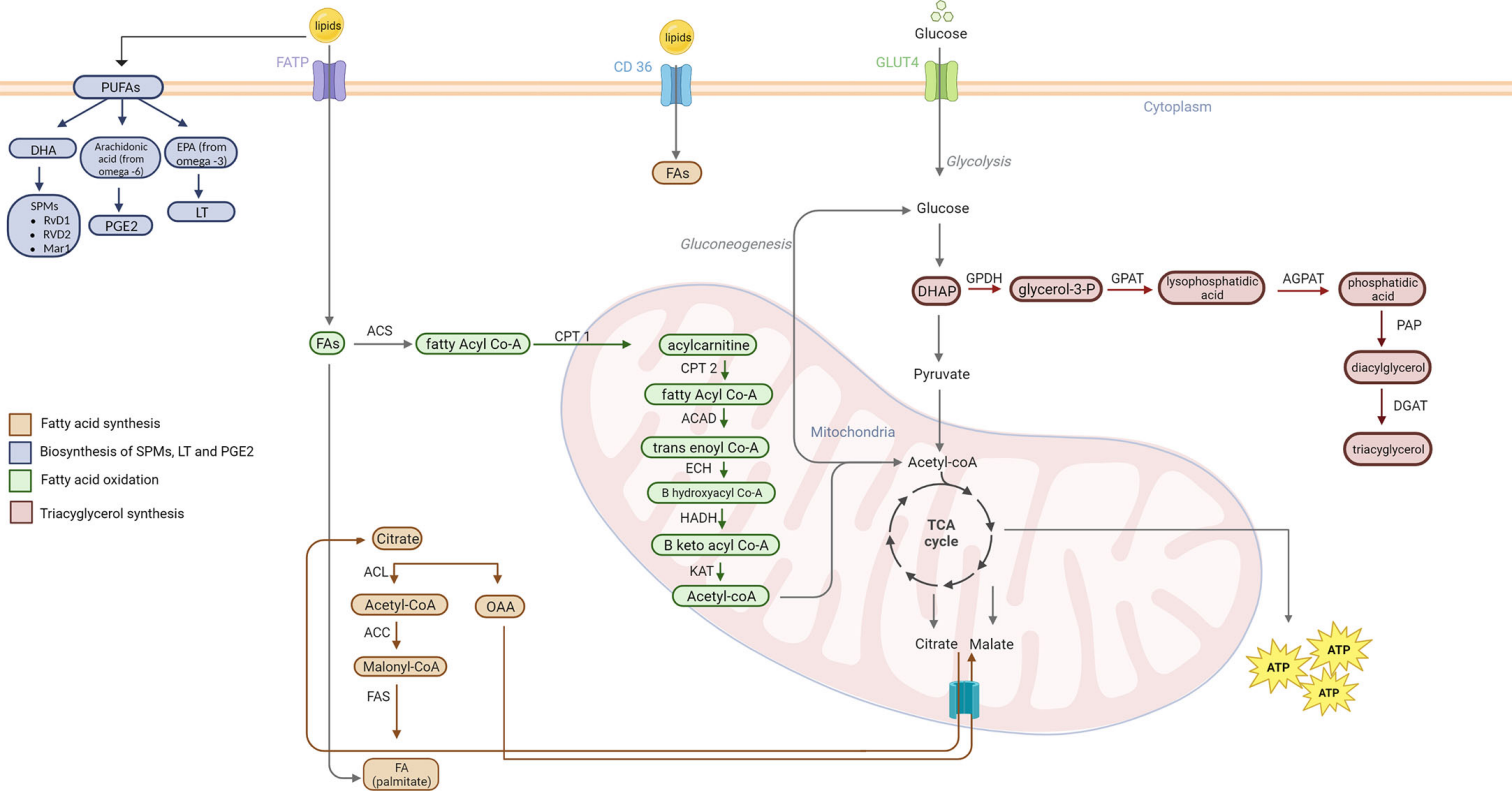
Lipid metabolism pathways. Fatty acid synthesis occurs when there is excess acetyl-CoA in the mitochondria. Acetyl Co-A combines with OAA to form citrate and is shuttled out of the mitochondria to the cytoplasm. ACL converts citrate back into acetyl-CoA. AAC activates acetyl-CoA to malonyl-CoA. FAS then combines the malonyl-CoA with another acetyl-CoA until palmitate is formed. Lipogenesis of cholesterol, phospholipids, and triacylglycerol also play a role in cancer pathogenesis. SPMs derive from DHA PUFA. EPA PUFAs from omega-3 FAs are used to create 5 series LT. Arachidonic acid from omega-6 FA are used for synthesis of PGE2. Triacylglycerol are formed from an intermediate in glycolysis, DHAP. The catabolic lipid metabolism, also called FAO, fatty acids are used to form acetyl-CoA for use in the TCA cycle. Fatty acyl-CoA is formed from FA *via* ACS. CPT1 (at the outer mitochondrial membrane) converts acyl-CoA to acyl-carnitine. CPT2 (at the inner mitochondrial membrane) then causes the release of carnitine and allows acyl-CoA to enter the mitochondrial matrix. ACAD catalyzes a dehydrogenation step to form trans-enoyl CoA. The following step is hydration *via* ECH that yields B hydroxyl CoA. B keto acyl CoA is formed by HADH mediate oxidation. Thiolysis of this molecule catalyzed by KAT forms acetyl-CoA which enters the TCA. PUFA, poly-unsaturated fatty acids; DHA, Docosahexaenoic acid; RvD1, Resolvin D1; RvD2, Resolvin D2; Mar1, Maresin1; PGE2, prostaglandin 2; EPA, eicosapentaenoic acid; LT, leukotrienes; FATP, fatty acid binding protein; FA, fatty acid; ACS, acetyl-CoA synthetase; CPT1, carnitine palmitoyl transferase I; CPT2, Carnitine palmitoyl transferase II; ACAD, acyl-CoA dehydrogenase; ECH, enoyl-CoA hydratase; HADH, hydroxyacyl-Coenzyme A dehydrogenase; KAT, ketoacyl-CoA thiolase; ACL, ATP citrate lyase; ACC, Acetyl-CoA carboxylase; FAS, fatty acid synthase; OAA, oxaloacetate; DHAP, Dihydroxyacetone phosphate; GPDH, glyceraldehyde 3-phosphate dehydrogenase; GPAT, Glycerol-3-phosphate acyltransferase; AGPAT, 1-acylglycerol-3-phosphate-O-acyltransferase; PAP, phosphatidate phosphatase, DGAT, Diglyceride acyltransferase.

Lipid metabolism is the biosynthesis and degradation of lipids in the cell. Lipids can be either consumed or synthesized *de novo* in the liver or adipose tissue (74). Understanding the mechanism behind lipogenesis and FAO can provide insight on possible therapeutic targets that regulate lipid metabolism (**Figure 3**).

Fatty acid synthesis occurs primarily in the liver and adipose tissues using excess glucose and amino acids (74). Acetyl Co-A is an intermediate in the tricarboxylic acid cycle (TCA) that is used in fatty acid synthesis. Acetyl-CoA is combined with oxaloacetate (OAA) to form citrate and leaves the mitochondria through the citrate-malate antiport to the cytoplasm where fatty acid synthesis occurs (74). Once in the cytoplasm, ATP citrate lyase (ACL) cleaves the citrate back into acetyl-CoA and OAA. Acetyl-CoA is activated to malonyl CoA by acetyl CoA carboxylase. Malonyl CoA is then used to form FA via fatty acid synthase (FAS).

Triacylglycerols (TAGs) are another important class of lipids used to store excess fat (74). TAG synthesis takes place in the smooth endoplasmic reticulum (ER) of adipose tissue and hepatocytes. Biosynthesis of TAGs begins with glycerol-3-phosphate (G3P) which is created in the liver by glycerol kinase or through reduction of dihydroxyacetone phosphate (DHAP), a glycolytic intermediate, by glycerol-3-phosphate dehydrogenase (GPDH) (74). Lysophosphatidic acid (LPA) is then produced through an acylation reaction catalyzed by sn-1-glycerol-3-phosphate acyltransferase (GPAT). LPA is then used to produce phosphatidic acid (PA) by acyl-CoA:1-acylglycerol-3-phosphate acyltransferase (AGPAT). PA is hydrolyzed to form diacylglycerol (DAG) by PA phosphatase (PAP) and DAG is subsequently esterified to produce TAG via DAG acetyltransferase (DGAT) (75).

In order to utilize the energy stored in lipids, cells undergo FAO (74). FAO primarily occurs in the mitochondria of hepatocytes. FAO begins with the conversion of fatty acyl-CoA by acyl-CoA synthetase (ACS). Fatty Co-A is then translocated into the mitochondria via the carnitine shuttle consisting of carnitine palmitoyl transferase I (CPT I) and CPT II. CPT I catalyzes the conversion of fatty acyl Co-A to acylcarnitine which is then used to reform fatty acyl-CoA via CPT II inside the mitochondria. Fatty acyl Co-A undergoes an oxidation reaction to produce trans enoyl Co-A catalyzed by acyl Co-A dehydrogenase (ACAD). A hydration reaction follows in which trans enoyl Co-A is used to create β hydroxyacyl Co-A via enoyl Co-A hydratase (ECH). β hydroxyacyl Co-A is then oxidized by β hydroxyacyl dehydrogenase (HADH) to produce β ketoacyl Co-A. β ketoacyl Co-A then undergoes a thiolysis reaction via ketoacyl-CoA thiolase (KAT) to form acetyl Co-A which can be used in the TCA (74).

The metabolism of lipids is highly regulated in the body. PPARα, PPARγ, SREBPs and carbohydrate response element binding protein (ChREBP) are key transcription factors that modulate FA synthesis in the body (76). PPARα is activated by FAs and reduces triacylglycerol levels in the blood via upregulation of lipoprotein lipase activity and FAO (76). PPARγ is involved in adipose tissues and contributes to increase triacylglycerol synthesis and lipid accumulation (77). Lipid metabolism can also be regulated in response to glucose via ChREBP (78). SREBPs regulate lipid metabolism by controlling the expression of enzymes required for lipogenesis (79). Given their influence on lipid metabolism, these transcription factors can serve as potential targets in lipid mediated immunotherapies.

### Role of lipid metabolism in cancer

As discussed in the previous section, lipids and their metabolism have significant roles in immune function and responsiveness. Lipids are also essential components of cell membranes and are important in cellular processes such as signaling, energy storage, and immune system function, meaning that lipids and lipid metabolism can influence the effectiveness of immunotherapies. As one example, having non-small cell lung cancer with a high mutation burden in the lipid metabolism pathway has been linked with better response to immune checkpoint therapy and prolonged progression-free survival (80). Another group found that T-cell senescence caused by tumor cells or Tregs in the TME could be reversed by reprogramming lipid metabolism. Specifically, unbalanced lipid metabolism related to senescence was found to elevate group IV A phospholipase A2, pharmacologic inhibition of which enhanced antitumor immunity in melanoma and breast cancer mouse models treated with adoptive T-cell transfer therapy (81).

Cancer cells can also “hijack” metabolic pathways to meet the increased demand for energy. For example, the conversion of FAs to phospholipids provides signals that activate proteins and bind to G protein-coupled receptors; this process enhances the proliferation, survival, and migration of malignant cells to establish distant metastases (82). As noted previously, malignant cells also consume FAs to sustain energy and promote their survival and use lipids to support their cellular membranes as well (82).

Other groups studying FA metabolism in cancer found that overexpression of acyl-CoA synthetase and stearoyl-CoA desaturase-1 prompted the epithelial-to-mesenchymal transition in colorectal cancer cells (83). Moreover, adipose tissues can release free FAs and secrete growth factors and cytokines after lipolysis. Indeed, in one study, co-culture of ovarian cancer cells with adipocytes led to activated lipolysis and released free FAs which in turn contributed to tumor-cell proliferation and migration (84). Thus, malignant cells can promote their survival and metastasis *via* lipid metabolism. Upregulation of lipogenic enzymes such as lysophosphatidic acid in various types of cancer cells has also been found to promote the growth of those cells (85).

## Lipids and cancer metastasis

The appearance of metastatic disease carries a poor prognosis for patients with cancer (86). Lipid accumulation and increased lipid production in cancer cells have been shown to increase metastasis. Different therapies to address FA-synthesizing enzymes have been explored in attempts to mitigate the tumor-cell migration. This topic remains largely unexplored, and additional research is needed to better understand the mechanism of by which lipids influence metastasis and how they affect therapies.

Two enzymes with key roles in lipid metabolism, FA synthase and monoacylglycerol lipase, participate in lipid synthesis (87, 88). In one study involving a model of prostate cancer in BALB/c mice, the metastatic potential of the cancer cells was found to increase when either enzyme was expressed in the presence of FA-binding protein 5. Specifically, expression of FA synthase and monoacylglycerol lipase led to increased prostate cancer cell migration and invasion. Conversely, treating these cells with C75, a FA synthase inhibitor, led to decreased migration and invasion compared with the control (89). Another enzyme involved in synthesizing triglycerides has also been linked with increased metastasis in gastric cancer. Specifically, mice fed a high-fat diet showed overexpression of diaglycerol acyl transferase 2 (DGAT2); when those mice were implanted with gastric cancer cells, the overexpression of DGAT2 led to increased peritoneal metastasis. Conversely, treating the mice with the DGAT2 inhibitor PF-06424439 suppressed mesenteric metastasis (86). Another series of experiments with an ovarian cancer model showed that overexpression of FA-binding protein 4 (FABP4) enhanced cancer cell proliferation via transfer of FAs from adipocytes (90, 91),; conversely, downregulation of FABP4 led to the formation of fewer metastatic nodules (90). Another group of lipids, the eicosanoids, have also been linked with inflammation and cancer progression. In a mouse model of colorectal cancer, exposure to the eicosanoid PGE2 increased the number of cancer stem cells and resulted in increased liver metastasis, which was found in mechanistic studies to be due to activation of nuclear factor κB in the EP4-MAPK and EP4-PI3K-Akt signaling pathways (92). Another type of eicosanoid, leukotrienes, have been implicated in priming the TME towards inflammatory (premetastatic) conditions (93); another group showed that leukotriene treatment promoted the epithelial-to-mesenchymal transition, thereby enhancing the capacity of the cells to migrate and metastasize (94).

The effects of lipids on cancer progression can also be assessed by studying transcription factors responsible for lipid production. Sterol regulatory element-binding protein 1 (SREBP-1) is a nuclear transcription factor responsible for the regulation of cholesterol, FA, and phospholipid synthesis. SREBP-1 has been shown to promote the gene transcription for three enzymes: FA synthase, acetyl-CoA carboxylase, and 3-hydroxy-3-methylglutaryl-CoA reductase. SREBP-1 is an important regulator of hepatocellular carcinoma; tissue samples from patients with hepatocellular carcinoma with upregulated SREBP-1 were associated with poor outcomes, and corresponding *in vitro* experiments showed that downregulation of SREBP-1 led to increased numbers of apoptotic cells and inhibited cell proliferation (95). These investigators concluded that SREBP-1 could be a potential therapeutic target in hepatocellular carcinoma.

## Lipids in cancer immunotherapy

### Lipids as adjuvants

Lipids have essential functions in cancer immunotherapy and can contribute directly or indirectly to therapy outcomes. Lipids are often used in cancer immunotherapy as adjuvants, that is, substances that augment an immune response by enhancing tumor-associated antigen presentation or activating antigen-presenting cells. One example of this is the use of adjuvants with tumor-associated-antigen subunit–based vaccines that elicit only weak immune response on their own. Among the many available cancer vaccine adjuvants are TLR4 and TLR7/8 agonists, which induce robust activation of antigen-presenting cells, CD4^+^ and CD8^+^ T cells, and NK cells, and shift the TME toward an inflammatory state through the expression of cytokines and chemokines (96). Moreover, lipid adjuvants can be combined with prophylactic or therapeutic cancer vaccines to enhance the effectiveness of those vaccines. One example is monophosphoryl lipid A, a modified form of a lipid presents in an endotoxin from Gram-negative bacteria that activates TLR4 and stimulates an inflammatory response (97). The adjuvant AS04, which contains monophosphoryl lipid A and alum, has been used successfully in Cervarix, a vaccine to prevent human papillomavirus (HPV) -16 and -18 –associated cervical cancer. The inclusion of the ASO4 adjuvant in this vaccine has been shown to evoke a more robust immune response in vaccinated people (98). Other synthetic lipid adjuvants such as 3M-052, GLA-SE, CRX-527, Ono-4007, OM-174, and DT-5461 have also been developed and applied for this purpose (99–103). Despite their somewhat limited clinical efficacy on their own, incorporating lipids as adjuvants in cancer therapy has promising potential, especially for tumors of low immunogenicity. Further study is required to improve their effectiveness and define their mechanistic effects on immunotherapies.

### Lipids as vehicles

Lipids are also used in nanoparticle form, including liposomes, solid lipid nanoparticles, and nanostructured lipid carriers, as a drug delivery vehicle to facilitate the transport of therapeutic agents to cancer cells and enhance their effectiveness. Coating drugs with lipid nanoparticles can increase drug bioavailability, biocompatibility, and biodegradability; reduce side effects; enable controlled release and extended periods in circulation; offer protection from chemical or enzymatic degradation; avoid the hepatic first-pass effect; and bypass the blood-brain barrier. Liposomes or lipid nanoparticles can carry both hydrophilic and lipophilic drugs, including immunomodulators, which can be loaded by mixing, conjugating, or encapsulating them into lipid particles to increase their efficacy. These nanoparticles can be administered via various routes, including topical, oral, parenteral, ocular, pulmonary, and intracranial (104). Further, coupling antibodies to the surface of the liposomes, thereby creating immunoliposomes, can enable and increase the specificity of targeted therapies (105).

Lipid nanoparticles are widely used to enhance antitumor responses by increasing the immunogenic effects of cancer immunotherapy. One example is resiquimod, a hydrophobic agonist of the Toll-like receptor (TLR) -7/8 used as an adjuvant in topical preparations for skin carcinomas; it has also been made more water-soluble by combining it with lipid particles and administering the compound systemically, to increase the efficacy of immunotherapy (106). Another group also showed that a liposomal preparation of resiquimod improved the adjuvant’s pharmacokinetics and prolonged its retention time in the blood, which also improved the effectiveness of immunotherapy (107). CpG oligodeoxynucleotides are another type of immune stimulator and vaccine adjuvant that act as a TLR9 agonist; their effectiveness can be increased by combining them with a liposomal nanoparticle carrier to enhance their delivery to macrophages and other immune cells after the initiation of antitumor response (108). Mifamurtide is a liposomal formulation of an immunomodulator used to treat osteosarcoma; it contains bacterial cell wall peptides that trigger the innate immune system to modulate an antitumor effect via NOD2 receptors (109).

Another novel way to use lipid nanoparticles in cancer immunotherapy is by loading them with tumor-specific or tumor-associated antigens to develop cancer vaccines. However, even though encasing these antigenic peptides in liposomes can reduces their degradation, increase their presentation to antigen-presenting cells, and stimulate CD4^+^ T-cell response to tumors, generating peptide vaccines is expensive and time-consuming. Therefore, mRNA vaccines have emerged as a better alternative because they are cheaper, well-tolerated, faster to produce on a large scale, and easier to combine with liposomes (110). After an mRNA vaccine is injected, cells take up the mRNA and begins the translation of desired proteins in the cytoplasm. These proteins in turn are split into peptides and then enter the MHC presentation cascade. Lipid nanoparticles can drastically enhance the effectiveness of mRNA vaccines by facilitating uptake of the mRNA and offering protection from degradation (111). The advent of mRNA vaccines encapsulated in lipid particles has led to many clinical trials for various types of cancer, including melanoma, glioblastoma ovarian, breast, gastrointestinal, genitourinary, hepatocellular, head and neck cancer, and lymphoma (112). Promising results from some of these trials provide the impetus for further investigation.

### The role of lipids in immune responsiveness

Lipids are essential components of the cell membrane and play a role in critical cellular processes, including signaling, energy storage, and immune system function. In the context of cancer immune therapy, lipids play critical roles in immune responsiveness and immune cells’ lipid metabolism in the tumor microenvironment is reprogrammed according to their unique needs and survival adaptations by increasing lipid uptake or *de novo* lipid synthesis. Lipid uptake is facilitated via transport proteins, including CD36, fatty acid transport proteins (FATPs), FABP or low-density lipoprotein receptors (LDLR) (113). For instance, intratumoral Tregs are shown to alter their lipid metabolism to increase their survival via the CD36- PPAR-β axis for metabolic adaptation to the tumor microenvironment by enhancing fatty acid transport and mitochondrial fitness. Targeting CD36 induces selective intratumoral Treg apoptosis due to metabolic stress in the microenvironment and contributes to immune checkpoint therapy (16). In another study, CD36 also facilitates ferroptosis, a regulated cell death mediated by iron-dependent lipid peroxidation in CD8+ T cells and reduces cytotoxic cytokine production. Blocking CD36 on CD8+ increases antitumor response and immune checkpoint therapy effect (17). It is also shown that fatty acid binder protein 2 (FATP2) is responsible for reprogramming pathological neutrophils called myeloid-derived suppressor cells (MDSc) via upregulation of arachidonic acid metabolism synthesis of PGE2. Pharmacologically targeting FATP2 diminished the evasive effect of MDSCs and tumor growth by reducing reactive oxygen species (ROS) and PD-L1 expression on tumor-infiltrating CD8+ T-cells (114, 115). Targeting *de novo* lipid synthesis is also promising in cancer immunotherapy to improve immune responsiveness. Tregs in the tumor microenvironment are responsible for immune evasion and it is shown that SREBs are responsible for *de novo* lipid synthesis and are required for the functional integrity of Tregs (116). Tregs also push M2 macrophage polarization and increase the M2 macrophage’s metabolic fitness, mitochondrial integrity and survival via *de novo* lipid synthesis. Targeting M2 macrophage survival by blocking *de novo* fatty acid synthesis via SREBP1 inhibitors in Tregs improves antitumor immunity and the efficacy of immune checkpoint therapy (117). Furthermore, elevated fatty acid synthase (FASN), a crucial enzyme in *de novo* lipid synthesis, confers more aggressive phenotypes to ovarian cancer. It is reported that high FASN expression diminishes tumor-infiltrating dendritic cells’ antigen-presenting capacity and blunts T cell-dependent antitumor immunity in mouse models. Adding FASN inhibitors partially restores immune response (118). Another study also showed supporting findings that lipid accumulation in dendritic cells causes functional dysfunction of dendritic cells that leads to immune evasion and pharmacological normalization of lipid levels via acetyl CoA carboxylase inhibitor augment cancer vaccine efficacy by restoring dendritic cell function (63).

It is shown that PD-1 expression in T cells, which is an exhaustion marker, alters T cell metabolism by increasing lipid metabolism and fatty acid oxidation (119). Fatty acid oxidation is also enhanced in Tumor-infiltrating MDSCs and FAO inhibition reduces the inhibitory effect of MDSCs and decreases their inhibitory cytokine productions (120). In melanoma models, it is shown that paracrine signaling enhances fatty acid oxidation via the β-catenin/PPAR-γ pathway in dendritic cells and induces the tolerization of the local dendritic cells. Blocking fatty acid oxidation with etomoxir reverses this immune-tolerant environment in the melanoma model and increases the immune checkpoint therapy effect (121). On the other hand, it is reported that exhausted CD8+ T cells enhance fatty acid catabolism to keep their function. Furthermore, augmenting fatty acid metabolism with peroxisome proliferator-activated receptor PPAR-α signaling activator agonist fenofibrate improves CD8+ function *in vitro* melanoma models and synergizes with immune checkpoint therapeutic effect (67).

In summary, promising evidence of correlations between lipids and cancer progression and metastasis is tantalizing, but much more research is needed to elucidate the mechanisms underlying these observations and apply them to anticancer therapy. Lipids are known to have profound effects on the immune system, and thus are candidate targets in immunotherapy to address cancer progression. However, lipids are also an important component of normal cellular functions, and targeting this biomolecule will require considerably deeper understanding of the mechanisms by which the immune cells are affected. The contradictory findings obtained to date on lipids and cancer progression could be attributable to the diversity of lipids, variations in different cellular contexts, and whether the experiments were conducted *in vitro* or *in vivo*. Further understanding of this important biomolecule can, it is hoped, prompt the development of novel lipid-targeted treatment approaches for cancer.

## Author contributions

LD and MC conceptualized and developed this review. LD, HC, TR, SG, SN, TV, and HB collected, analyzed, and interpreted the relevant literature. JW and MC critically reviewed the manuscript. All authors contributed to the article and approved the submitted version.
