# Corrigendum: Lipid metabolism in tumor immunology and immunotherapy

**DOI:** 10.3389/fonc.2023.1358498

**Published:** 2024-01-08

**Authors:** Lisa K. Duong, Halil Ibrahim Corbali, Thomas S. Riad, Shonik Ganjoo, Selene Nanez, Tiffany Voss, Hampartsoum B. Barsoumian, James Welsh, Maria Angelica Cortez

**Affiliations:** ^1^ Department of Radiation Oncology, The University of Texas MD Anderson Cancer Center, Houston, TX, United States; ^2^ Department of Medical Pharmacology, Cerrahpasa Medical Faculty, Istanbul University-Cerrahpasa, Istanbul, Türkiye

**Keywords:** lipids, lipid oxidation, immunotherapy, cancer, immune cells

In the published article, an author name was incorrectly written as Hampartsum Barsomium. The correct spelling is Hampartsoum B. Barsoumian.

In the published article, there was an error in the author contribution list, and author’s initials HB was erroneously excluded. The corrected author list appears below.

LD and MC conceptualized and developed this review. LD, HC, TR, SG, SN, TV, and HB collected, analyzed, and interpreted the relevant literature. JW and MC critically reviewed the manuscript. All authors contributed to the article and approved the submitted version.

The authors apologize for these errors and state that these do not change the scientific conclusions of the article in any way. The original article has been updated.

